# Nucleic Acid Scavenging Polymers Inhibit Extracellular DNA-Mediated Innate Immune Activation without Inhibiting Anti-Viral Responses

**DOI:** 10.1371/journal.pone.0069413

**Published:** 2013-07-23

**Authors:** Eda K. Holl, Kara L. Shumansky, George Pitoc, Elizabeth Ramsburg, Bruce A. Sullenger

**Affiliations:** 1 Department of Surgery, Duke University, Durham, North Carolina, United States of America; 2 Duke Translational Research Institute, Duke University, Durham, North Carolina, United States of America; 3 Department of Medicine, Duke University, Durham, North Carolina, United States of America; Massachusetts General Hospital and Harvard University, United States of America

## Abstract

Toll-like receptor (TLR) family members, 3, 7 and 9 are key components in initiation and progression of autoimmune disorders such as systemic lupus erythematosus (SLE). These TLRs are often referred to as nucleic acid-sensing TLRs based on their ability to recognize DNAs or RNAs produced by pathogens or damaged cells. During autoimmune disease progression these receptors recognize self nucleic acids as well as self nucleic acid-containing complexes and contribute to inflammatory cytokine production and subsequent enhancement of serum autoantibody levels. We have recently discovered that nucleic-acid scavenging polymers (NASPs) can neutralize the proinflammatory effects of nucleic acids. Here, we begin to explore what effects such NASPs have on normal immune function. We show that such NASPs can inhibit TLR activation without affecting nucleic acid-independent T cell activation. Moreover, we observe that stimulation of immune cells by encapsulated nucleic acids, such as those found in viral particles, is unaffected by NASPs. Thus NASPs only limit the activation of the immune system by accessible extra-cellular nucleic acid and do not engender non-specific immune suppression. These important findings suggest that NASPs represent a new approach toward anti-inflammatory drug development as these agents can potentially be utilized to block overt autoimmune disorders and inflammation while allowing normal immune responses to occur.

## Introduction

Toll-like receptors (TLRs) have been extensively characterized as initiators of innate and adaptive immune responses to pathogenic stimuli. Mammalian TLRs are found on the cell surface as well as in the endosomal compartment of various immune cells. Cell surface TLRs recognize different bacterial or viral products including LPS whereas endosomal TLRs recognize nucleic acids derived from microbes [1]. Stimulation of TLRs results in the initiation of a cascade of inflammatory responses characterized by the activation of transcription factors, such as the nuclear factor of light polypeptide gene enhancer in B cells 1 (NF-kB) as well as proinflammatory cytokines such as type 1 interferons (IFNs) [2].

Endosomal TLRs 3, 7, 8 and 9 are essential in controlling viral and bacterial infections by sensing non-self nucleic acids [3]–[5]. TLR7 recognizes specific sequences in guanosine- and uridine-rich ssRNA, whereas TLR3 and TLR9 sense dsRNA and unmethylated CpG motifs in dsDNA, respectively [5]–[8]. TLR8 recognizes viral ssRNA and controls IFN production, in humans [7], [9]. However, when circulating immune complexes that contain self-nucleic acids reach the endosomal compartment they can cause inappropriate activation of TLRs [10]. Although mechanisms exist to prevent activation of TLRs by self nucleic acids, initiation of aberrant immune responses commonly occurs due to insufficient repression. For example, self-nucleic acids released by dying cells can complex with other cytosolic proteins such as the high mobility group box (HMGB1) and activate endosomal TLRs [11]. This response in turn can contribute to the activation of the inflammatory cytokine signaling cascade and subsequently enhancement of autoimmune diseases [10], [11].

Many complex autoimmune disorders are thought to be initiated by inappropriate activation of immune cells via self nucleic acids and nucleic-acid immune complexes [12]. For example, a number of immune cells including plasmacytoid dendritic cells (pDCs) and B cells have been shown to play an important role in systemic lupus erythematosus (SLE) autoimmune disease onset and progression due to their ability to produce pro-inflammatory cytokines and self-reactive antibodies [13]. Upon activation, pDCs rapidly produce large amounts of type I interferons (IFNs) which then lead to conventional DC (cDC) maturation and further pro-inflammatory cytokine production [13], [14]. TLR activation of cDCs themselves also results in cell maturation, cytokine production and subsequent T cell activation [13]. Self-nucleic acid TLR ligands can contribute to B cell activation during autoimmune disease development [15]–[17]. This activation in turn results in production of pathogenic antibodies. Similarly, autoimmune disorders such as multiple sclerosis and rheumatoid arthritis have been shown to be dependent on DC or T cell activation and TLR stimulation [18], [19]. TLR ligands have been used to trigger these organ specific autoimmune disorders and blocking the TLR negative regulators can result in spontaneous autoimmune disease development via induction of pro-inflammatory cytokine production such as type I IFNs [12], [20].

Blocking overt activation of endosomal TLRs by self-ligands is crucial in treating autoimmune disorders [21], [22]. Current therapies that have been shown to slow down SLE progression focus on direct inhibition of TLR7 and TLR9 via immunoregulatory DNA sequence (IRS) 954 [22]. For example, IRS954 treatment of lupus prone mice (NZBWF1) results in reduced autoantibody production and reduced glomerulonephritis [22]. Additionally, studies of animals lacking TLR7 and 9 genes in a lupus background have shown reduced disease onset and development [23]. Taken together these studies suggest that targeting of these particular TLRs is a key component in SLE treatment. Although somewhat successful, these therapies rely on directly blocking the function of two TLRs, 7 and 9. Given the importance of TLR7 and 9 in protection from viral and bacterial infections [7], [24], [25], long-term therapeutics, directly targeting TLR7 and 9 are expected to be detrimental to the host’s ability to combat such infections. Therefore, an alternative and potentially safer therapeutic approach would be to focus on scavenging pro-inflammatory self-nucleic acids prior to TLR binding, thus allowing normal immune responses to pathogenic nucleic acids to proceed through functional TLRs.

A previous publication from our laboratory reported that a number of NASPs are capable of binding ssRNA, dsRNA, and unmethylated DNA and preventing cell activation through endosomal TLRs [26]. To extend these studies and begin to determine if such NASPs have unwanted immune suppressive properties, we examined whether these NASPs cause non-specific or selective suppression of immune cell functions. Previous studies have reported that microbial and viral nucleic acids exist in a shielded state and thus, differ from endogenous pro-inflammatory nucleic acids [27]. Here we show that viral capsids protect these virulent nucleic acids from NASP binding and neutralization. These findings demonstrate that nucleic acid scavenging polymers may represent a new potential set of therapeutic agents to fight autoimmunity due to their ability to block TLR activation by nucleic acids without interfering with the normal course of an immune response.

## Materials and Methods

### Ethics Statement

All studies were conducted in accordance with the National Institutes of Health Guide for the Care and Use of Laboratory Animals and were approved by the Duke University Institutional Animal Care and Use Committee (protocol number A011-11-01).

### Mice

C57BL/6 and OT-II mice (B6.Cg.Tg(TcraTcrb)425Cbn/J), specific for the ovalbumin residue 323–339, were obtained from the Jackson Laboratory (Bar Harbor, ME). Mice were housed in a pathogen-free barrier facility at Duke University.

### Cationic Polymers

Hexadimethrine Bromide (HDMBr, Cat # 107689) and PAMAM-G3 (Cat# 412422)were obtained from Sigma-Aldrich. CDP was a kind gift from Dr. Mark Davis (California Institute of Technology). All polymers were resuspended in PBS prior to use.

### Cell Culture

#### B cell activation

B cells from spleens of C57BL/6 animals were purified by negative selection (STEMCELL). B cell purity was over 95%. 2×10^5^ purified B cells were cultured in RPMI media (Gibco) containing 10% FBS, 10^−4^M 2-ME and penicillin/streptomycin (P/S) antibiotics in 96 well plates. Cells were cultured in the presence of 100 ng/ml LPS (SIGMA) or 1 µM CpG Invivogen) plus 20 µg/ml CDP or PAMAM-G3 for 18 hours or 72 hours. Alternatively, B cells were labeled with CFSE and cultured as above for 48 hours. Proliferation was assessed using flow cytometry. *DC culture*: Murine bone marrow DCs were isolated C57BL/6 mice and were cultured in the presence of GM-CSF and IL-4 as previously described [28]. 1×10^5^ DCs were cultured in RPMI media (Gibco) containing 10% FBS, 10^−4^M 2-ME and penicillin/streptomycin (P/S) antibiotics in 96 well plates. Cells were cultured in the presence of 100 ng/ml LPS (SIGMA) or 1 µM CpG-1668 Invivogen) plus 20 µg/ml CDP or PAMAM-G3 for 30 minutes, 1, 6 and 18 hours. p*DC culture*: Murine bone marrow was isolated from C57BL/6 mice and were cultured in the presence of Flt3 ligand for 10 days. Cells were then isolated and purified using a mouse pDC isolation kit (Mylteni) with purity assessed at >90%. 1×10^5^ pDCs were cultured in RPMI media (Gibco) containing 10% FBS, 10^−4^M 2-ME and penicillin/streptomycin (P/S) antibiotics in 96 well plates. *T cell culture:* T cells were obtained from OT-II mice and purified by negative selection (STEMCELL). *DC:T cell co-cultures:* DCs were harvested at day 7 and pulsed overnight with 10 mg/ml OVA (Sigma-Aldrich). 200,000 DCs were then washed and cultured in a 1∶10 ratio with CFSE-labeled T cells from OT-II transgenic T cells in 6 well plates in the presence of 20 µg/ml PAMAM-G3, HDMBr and CDP. *T cell restimulation:* 3 days post culture T cells were restimulated in the presence of 5 ng/ml PMA (Sigma) and 500 ng/ml Ionomycin (Sigma) for a total of 6 hours. BerfeldinA was added to cultures 4 hours prior to harvest. Cells were then stained for the presence of intracellular cytokines.

### Virus Assays

#### Viruses

Vesicular stomatitis virus (Indiana strain) was originally obtained from Dr. John Rose (Yale University). Virus was propagated on BHK cells and titered using a standard plaque assay on BHK cells. Vaccinia virus Western Reserve strain was obtained from BEI Resources. Virus was propagated and titered on HeLa cells using a standard plaque assay as described (Barefoot et al., Vaccine, 2008). *Virus Infections.* Bone marrow derived dendritic cells and plasmacytoid dendritic cells were plated on 24well tissue culture plates at a density of 1×10^6^ cells/well in 500 µLs complete RPMI media. Cells were infected with vesicular stomatis virus at an MOI of 5 or with vaccinia virus at an MOI of 10 in the presence or absence of 20 µg/mL PAMAM-G3. CpG 1668 and 1585 were used as controls for cell stimulation respectively. Cells were incubated at 37°C for 24 hours and supernatants were collected.

Bone marrow derived dendritic cells were stimulated with 100 ng/mL LPS, 1 µM CpG or VSV and treated with 0.5 µg/mL CLI-095 (TLR-4 inhibitor), 2 µM IRS954 and/or 20 µg/mL PAMAM-G3.

### ELISA

#### Antibody ELISA

ELISA plates (BD Falcon) were coated with 2 µg/ml (100 µl/well) of capture reagent goat anti-mouse Ig_(H+L)_ in 0.1 M Carbonate Buffer (pH 9.5) overnight at 4°C. HRP-conjugated goat anti-mouse IgM from Southern Biotechnology Associates (Birmingham, AL) was used as a detection antibody. Each cultured sample was diluted 1∶2 and was followed by 5 serial 2-fold dilutions and assayed in duplicate. Purified mouse IgM mAb was used to generate standard curves beginning at 1 µg/ml and diluted to 6 ng/ml.

#### Cytokine ELISA

Murine IL-6, TNFα and IFNα in cell culture media were quantified using Mouse IL-6 ELISA Ready-SET-Go (eBioscience), Mouse IFN alpha Platinum ELISA (eBioscience), or VeriKine™ Mouse Interferon-Alpha ELISA Kit (PBL) according to the manufacturers protocol. Each cultured sample was run as undiluted and then followed by 5 serial 2 fold dilutions and assayed in duplicate.

### Antibodies and FACS

Monoclonal Abs included: B220 (RA3-6B2); CD45.2 (104), CD4 (L3T4), CD8 (Ly-2), IFNγXMG1.2), IL2 (JES6-5H4), IL4 (11B11), CD3 (145-2C11), CD11c (N418) and TCRβ (H57-597) from Beckman Coulter (Miami, FL) and eBioscience (San Diego, CA). Single cell suspensions of cultured cells were counted and 10^6^ cells were suspended in FACS buffer (1×PBS plus 2% FBS) and stained with various antibody combinations. Flow cytometry was performed on a Gallios flow cytometer and FACSCanto. All data was analyzed with FlowJo software (Tree Star).

### Statistical Analysis

Statistical significance was determined with two-tailed Student’s t test or analysis of variance (ANOVA). All *p* values less than 0.05 were considered significant.

## Results

### Certain Nucleic Acid-binding Scavengers do not Inhibit Antigen Phagocytosis by Dendritic Cells, MHCII Presentation and T Cell Activation

**Figure 1:**
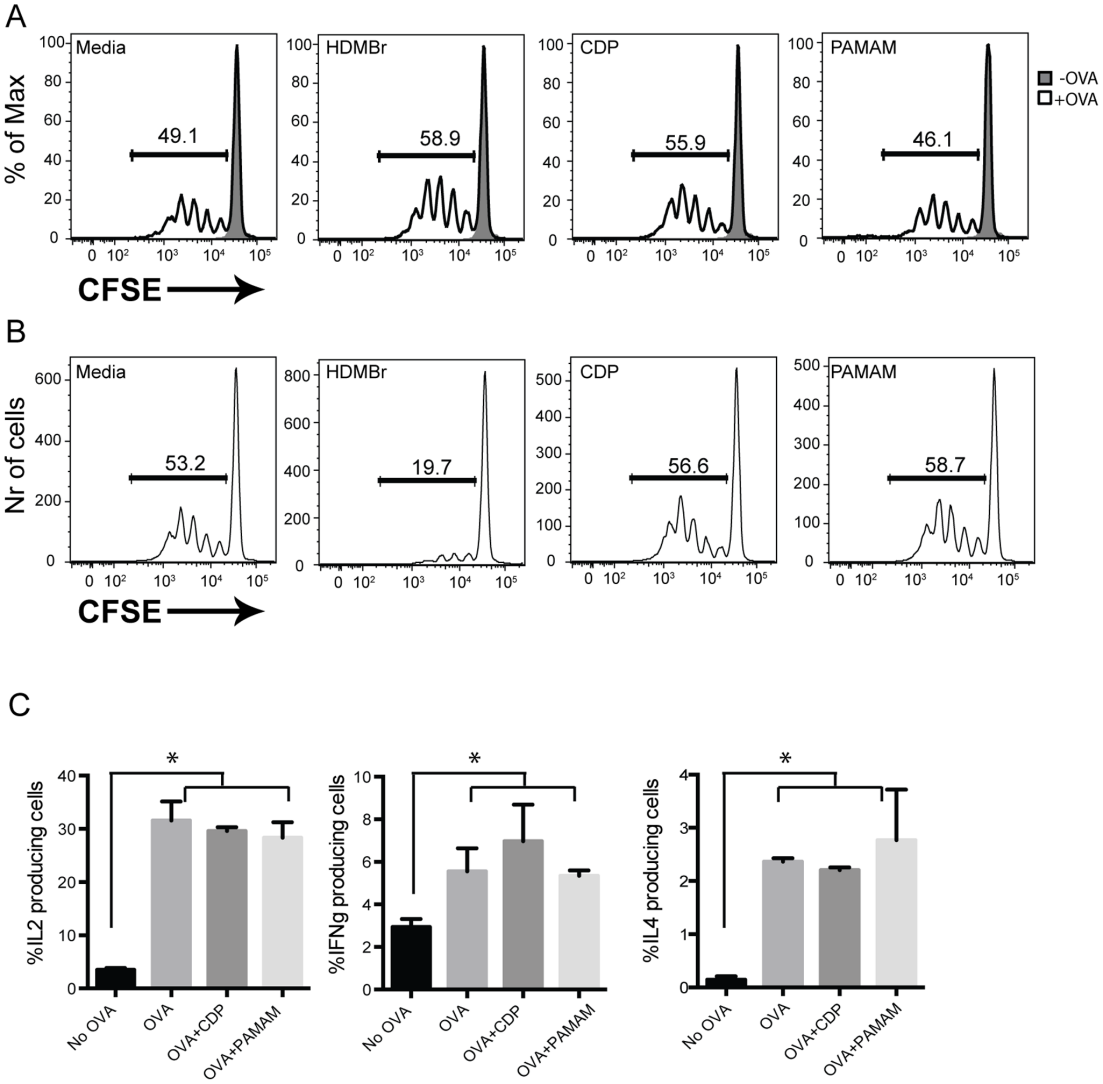
Certain nucleic acid binding scavengers do not inhibit antigen phagocytosis by DCs, MHCII presentation and T cell activation. (A) Polymers do not affect DC-driven T cell proliferation when cells are co-cultured with them simultaneously. DCs were derived in the presence of GM-CSF and IL4 from the bone marrow wild type mice. They were pulsed with OVA protein for 6 hours and were then co-cultured with CFSE-labeled OTII-specific T cells in the presence or absence of HDMBr, CDP and PAMAM-G3 for 3 days. T cell proliferation was assessed using CFSE dilution by flow cytometry. (B) Pretreatment of DCs with HDMBr results in impaired ability of DCs to stimulate T cells. DCs were grown as above. Cells were then cultured in the presence of 20 µg/ml HDMBr, CDP and PAMAM-G3. They were then washed and pulsed with OVA protein for 6 hours and cultured in the presence of CFSE labeled OTII-specific T cells. Proliferation of T cells was assessed as described above. (C) CDP and PAMAM-G3 polymers do not affect T-cell cytokine production post DC stimulation. DCs were cultured in the presence of 20 µg/ml CDP or PAMAM-G3. They were then washed and pulsed with OVA protein for 6 hours and cultured in the presence of OTII-specific T cells. Three days post culture; cells were stimulated with PMA and ionomycin for 6 hours in the presence of brefeldin A. Cells were then harvested and stained for the presence of intracellular cytokines. IL2, IL4 and IFNγ levels were assessed by flow cytometry. All data are representative of at least three independent experiments. *p<0.05.

DCs play an important role in priming naïve T cells in secondary lymphoid organs [29]. Aberrant DC-driven T cell activation can lead to initiation and maintenance of autoimmune disorders [13]. Ideal approaches to preventing and treating autoimmune disorders focus on blocking aberrant DC and T cell activation without interfering with normal DC-T cell responses. To test whether the NASPs non-specifically interfere with MHC-II presentation, BMDCs from WT mice were pulsed with ovalbumin (OVA) protein and then co-cultured with OTII transgenic T cells specific for OVA peptide 323–339. All cultures were performed in the presence of 20 µg/ml as this was the optimal dose determined for cell culture (Figure S1). Proliferation of OTII T cells was assessed by flow cytometry. DCs that were co-cultured with T cells in the presence of NASPs were able to stimulate T cells to a similar level as non-NASP treated cells (Figure 1A). These data suggest that the cationic polymers tested do not affect MHCII trafficking and antigen presentation when co-cultured with BMDC during antigen uptake.

We next assessed the effects that NASPs have on DC-driven T cell activation when DCs were treated with NASPs prior to OVA uptake. T cell activation was not affected in the presence of CDP and PAMAM-G3 (Figure 1B). However we did observe that pretreatment of BMDCs with one NASP, HDMBr, did result in impaired MHC-II presentation of OVA to OT-II transgenic T cells. Therefore, pretreatment of DCs with CDP and PAMAM-G3 but not HDMBr results in normal T-cell activation regardless of nucleic acid TLR stimulation. We have previously reported that cell viability is not affected by CDP, PAMAM-G3 or HDMBr at the experimental doses used here. Thus the reason for this MHC-II presentation difference is unclear and additional work will be required to elucidate the mechanism behind our observation. Nevertheless two of the three NASPs tested are fully supportive of MHC-II presentation. Therefore, from here on out we will focus on two NASPs, CDP and PAMAM-G3 and their effects on immune responses.

To further examine if NASPs have non-specific effects on effector T cell activation we assessed whether cytokine production occurs in response to NASPs treatment of DCs. To this end, IL2, IL4 and IFNγ levels were determined by flow cytometry. IL2, IL4 and IFNγ cytokine production by the activated T cells was not altered by CDP and PAMAM-G3- treated cells (Figure 1C). These results are consistent with the DC-induced T cell proliferation data (Figure 1A and 1B) and indicate that CDP and PAMAM-G3 have minimal impact on T cell activation. Therefore, future studies will likely focus on CDP and PAMAM-G3 polymers in order to avoid non-specific inhibition of immune cell activation.

### Pre-treatment of Cells with CDP and PAMAM-G3 does not Result in Impaired OVA-uptake

**Figure 2:**
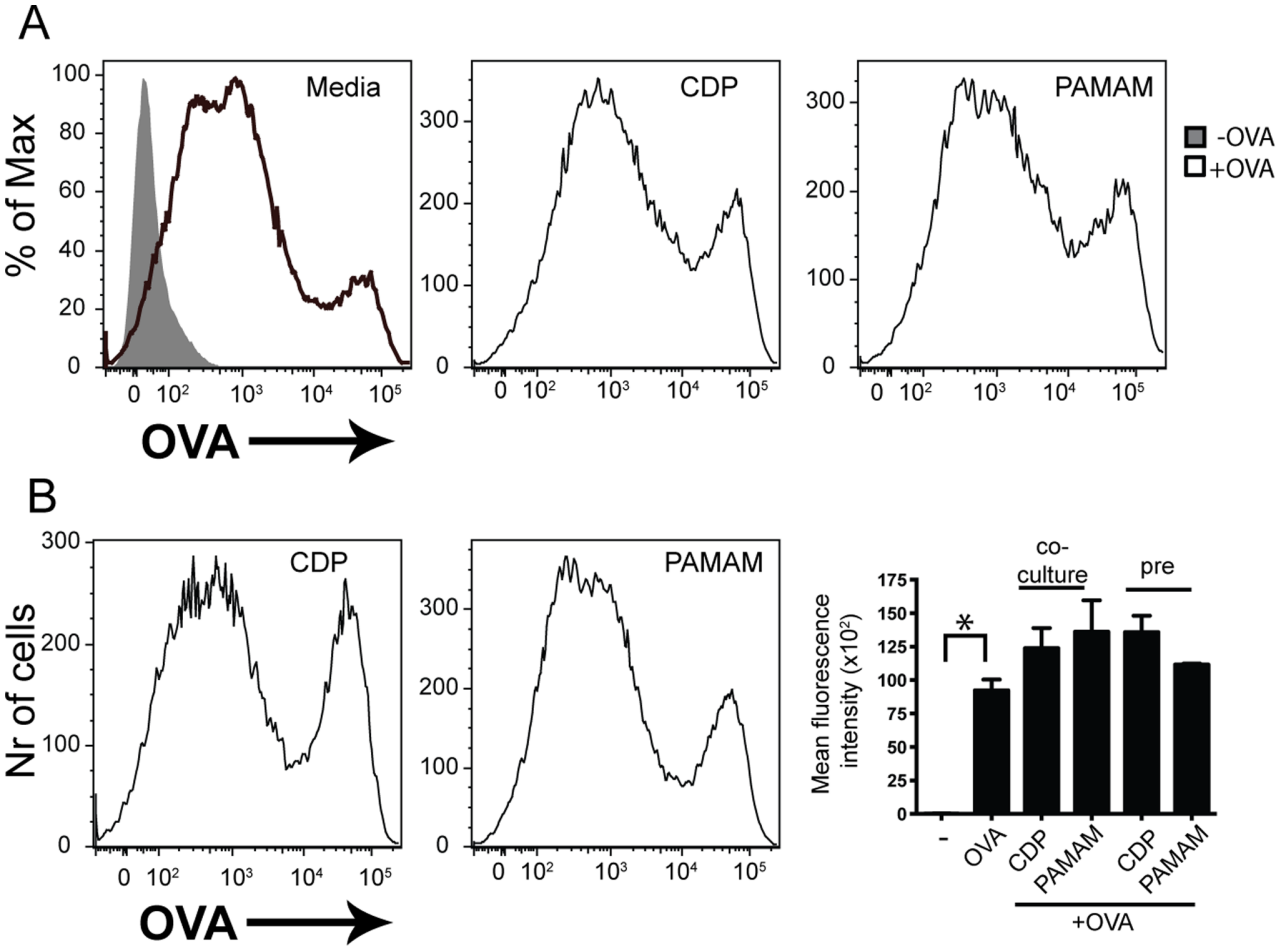
Pre-treatment of DCs with CDP and PAMAM does not affect T cell activation and proliferation by DCs. (A) Co-culturing of DCs with polymers does not result in impaired OVA uptake. BMDCs were cultured in the presence of 20 µg/ml CDP or PAMAM-G3 as well as 1 mg/ml OVA-FITC for 30 minutes. OVA uptake was then assessed by flow cytometry. (B) Pre-treatment of DCs with polymers does not affect the ability of DCs to uptake OVA antigen. BMDCs were cultured in the presence of 20 µg/ml CDP or PAMAM-G3. Cell were then washed and pulsed with OVA-FITC for 30 minutes. OVA uptake was then assessed by flow cytometry. Data are representative of at least three independent experiments. Data from three different experiments are compiled in MFI graph. *p<0.05.

We proceeded to examine whether antigen uptake was affected in the presence of NASPs before and during OVA stimulation. DCs induce T cell proliferation by presenting antigen in the context of MHC molecules. To determine the ability of DCs to take up antigen, we cultured them in the presence of FITC-labeled OVA protein and NASPs for 30 minutes. The amount of OVA taken up by the DCs was determined by flow cytometry. Our data indicate that NASPs do not directly affect antigen uptake by DCs as seen in representative plots (Figure 2A). Additionally, we determined whether pre-treatment of DCs with NASPs affected their ability to take up antigen. All mean fluorescence intensity data are compiled in a bar graph. Our data show that treatment of cells with CDP and PAMAM-G3 has no effect on antigen uptake (Figure 2B). This is an important observation given that our goal is to obtain potential therapeutic agents that do not exhibit non-specific immune suppressive effects.

### Co-stimulatory Molecule Expression in the Presence of NASPs

**Figure 3:**
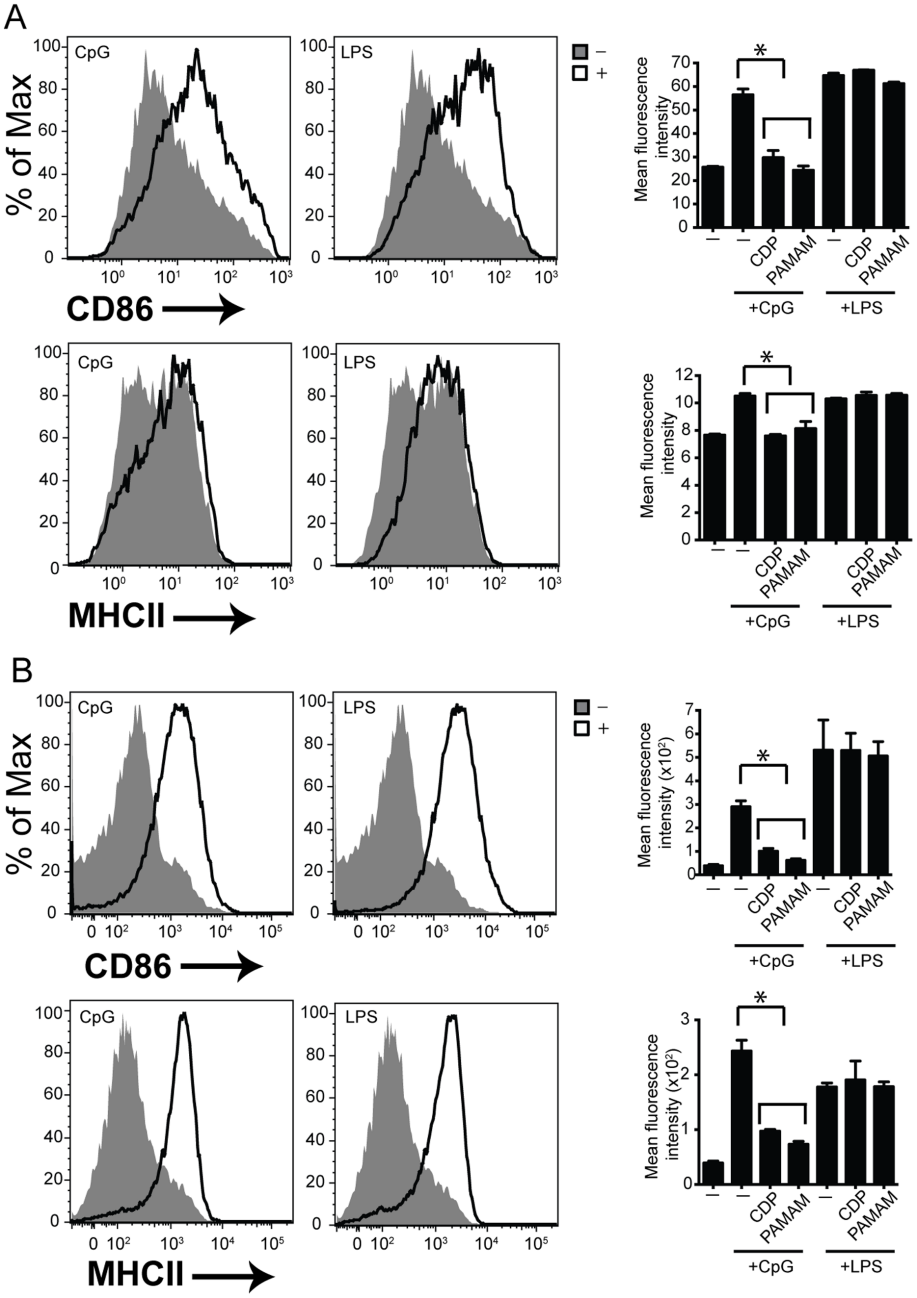
Polymers regulate cell surface receptor expression on B cells and DCs post nucleic acid stimulation. (A) Polymers block cell surface receptor expression post CpG but not LPS stimulation. BMDC were cultured as previously described. DCs were then cultured in the presence of LPS and CpG as well as 20 µg/ml of each polymer (CDP and PAMAM). CD86 and MHCII cell surface receptor expression was assessed 24 hours later using flow cytometry. Data are representative of 3 independent experiments. n = 9 mice per group. *p<0.05. (B) Polymers block cell surface receptor expression post CpG but not LPS stimulation. B cells were isolated and cultured as previously described. B cells were then cultured in the presence of LPS and CpG as well as 20 µg/ml of each polymer. CD86 and MHCII cell surface receptor expression was assessed 24 hours later using flow cytometry. Data are representative of 3 independent experiments. n = 9 mice per group. *p<0.05.

We next sought to determine whether MHCII and costimulatory molecule CD86 expression post TLR activation was affected in the presence of NASPs. TLR activation results in upregulated MHCII and costimulatory molecule CD86 expression. We stimulated C57BL/6 mouse DCs with TLR4 and TLR9 agonists in the presence of CDP and PAMAM-G3 and determined the ability of these cells to express cell surface MHCII and CD86 by flow cytometry. The mean fluorescence intensity (MFI) of cell surface molecules post stimulation was calculated and graphed. As compared to cells treated with CpG alone, cells treated with CDP or PAMAM and CpG DNA had reduced CD86 and MHCII cell surface expression. By contrast, the NASPs had no affect on cells that were activated by LPS through TLR4 (Figure 3A). These data indicate that polymers specifically block activation of cells via nucleic acid-based TLR agonists but not other non-nucleic acid-based TLR agonists.

We extended our studies to a different immune cell type important for mounting normal effective antiviral responses, B cells. C57BL/6 splenic B cells were stimulated similarly to DCs in the presence of TLR4 and TLR9 agonists. Cell surface MHCII and CD86 expression was assessed using flow cytometry (Figure 3B). These data indicate that CDP and PAMAM block MHCII and CD86 expression post nucleic acid activation of TLR9 but not activation via TLR4. Taken together these data indicate that NASPs block cell surface molecule expression post nucleic acid TLR but not non-nucleic acid TLR activation. These findings are consistent with our previous work [26] and further expand upon the role of NASPs in co-stimulatory cell surface expression across different cell types, some of which appear to be more sensitive to nucleic acid stimulation.

### Polymers Regulate B Cell Proliferation and Antibody Production

**Figure 4:**
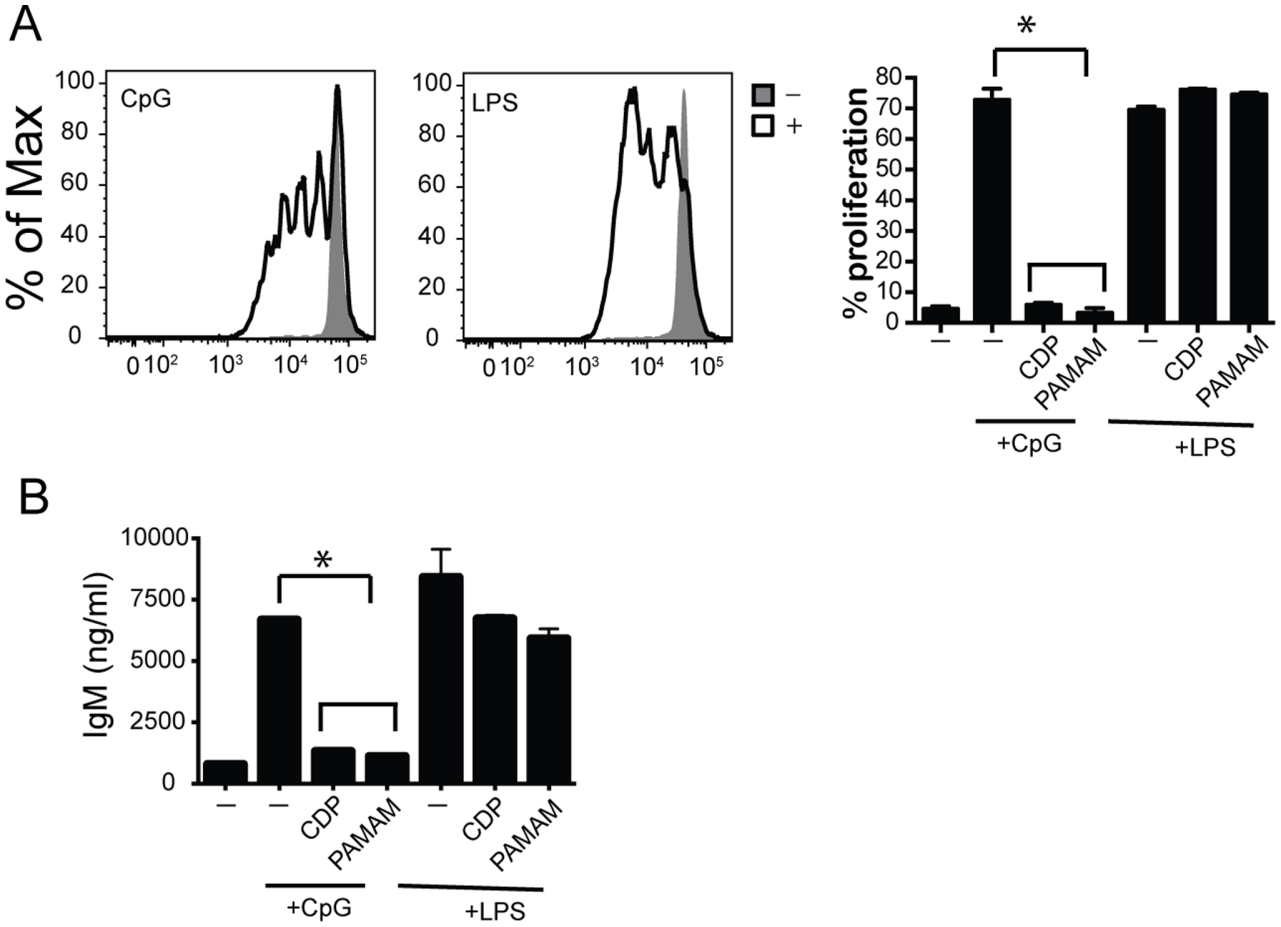
Polymers regulate B cell proliferation and antibody production post nucleic acid stimulation. (A) Polymers block B cell proliferation post CpG but not LPS stimulation. B cells were isolated and CFSE labeled. They were then cultured in the presence of LPS and CpG as well as 20 µg/ml of CDP or PAMAM polymer. Proliferation was assessed using CFSE dilution using flow cytometry. Flow plots show cell proliferation in the presence or absence of stimulation. Graph (right) complies proliferation data for stimulations in the presence or absence of polymers. Data are representative of 3 independent experiments. n = 9 mice per group. p<0.05. (B) Polymers block IgM antibody production post CpG but not LPS stimulation. B cells were cultured in the presence of CpG and LPS as well as 20 µg/ml of CDP or PAMAM polymer. Supernatants were collect 72 hours post stimulation and IgM levels were assessed via ELISA. Data are representative of 3 independent experiments. *p<0.05.

Aberrant rapid B cell proliferation and antibody production post nucleic acid stimulation of TLRs can lead to autoimmune disease development and maintenance [15]. To determine whether NASPs play a role in TLR-driven B cell proliferation and antibody production, splenic B cells were stimulated *in vitro* with TLR4 and TLR9 agonists in the presence of CDP and PAMAM-G3. Splenic B cells were isolated from C57BL/6 animals, labeled with carboxyfluorescein succinimidyl ester (CFSE) and stimulated with CpG and LPS. Rounds of proliferation were assessed by flow cytometry. Representative flow plots showing B cell proliferation post CpG or LPS stimulation are shown in Figure 4A. TLR9-driven B cell proliferation was abolished in the presence of NASPs (flow plots not shown). All of these data are compiled and presented in a graph (Figure 4A). B cell proliferation was unaffected post TLR4 stimulation in the presence of NASPs.

We next assessed antibody production by B cells that were stimulated with CpG and LPS. IgM production in the cultures was assessed 72 hours post stimulation via ELISA. NASPs blocked antibody production post TLR9 activation but not TLR4 activation in B cells (Figure 4B). Taken together these data suggest that the effects of NASPs on cell activation can go beyond initial cytokine production. This further suggests that NASPs block activation of immune cells by scavenging nucleic acids and limiting initial as well as long term cell activation.

### Effects of PAMAM-G3 Dendrimer on the Immune Response to Viral Infection

**Figure 5:**
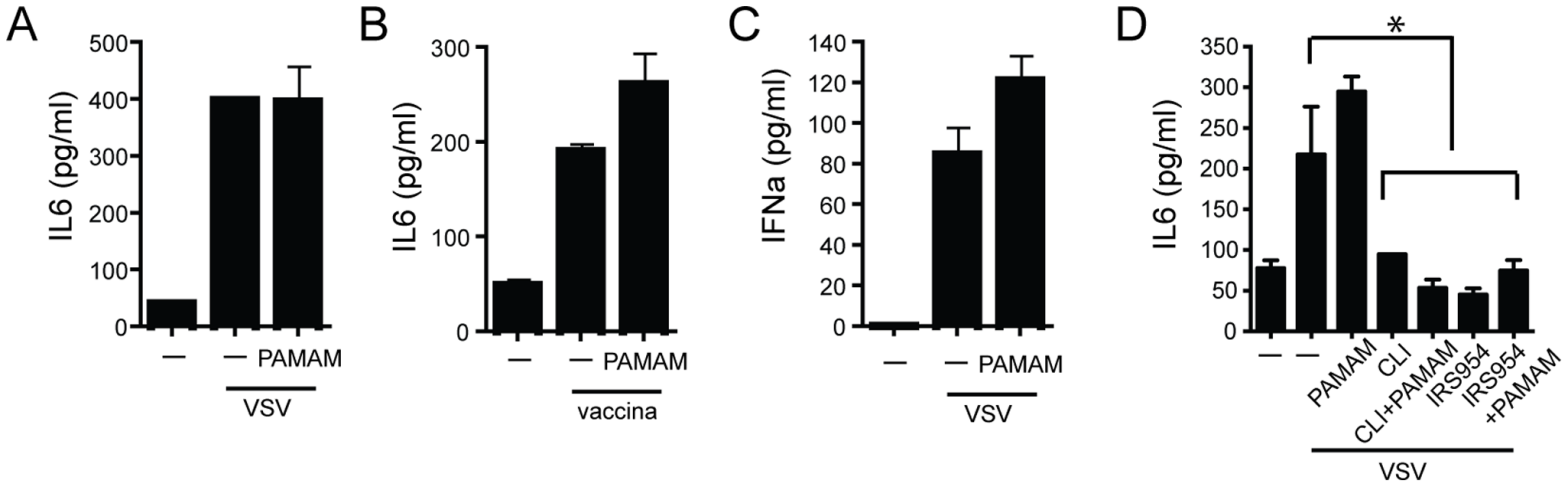
Polymers do not affect DC activation by encapsulated viruses. (A) Polymers do not affect TNFα production post VSV stimulation. DCs were generated form bone marrow cells of WT animals cultured in the presence of IL4 and GMCSF for 7 days. Cells were then stimulated with live VSV at a multiplicity of infection (MOI) of 1 in the presence of 20 µg/mL PAMAM-G3. Culture supernatants were analyzed for secretion of IL6 by ELISA, 24 h later. (B) Polymers do not affect TNFα production post vaccinia stimulation. DCs were generated form bone marrow cells of WT animals cultured in the presence of IL4 and GMCSF for 7 days. Cells were then stimulated with live vaccinia at a multiplicity of infection (MOI) of 1 in the presence of 20 µg/mL PAMAM-G3. Culture supernatants were analyzed for secretion of IL6 by ELISA, 24 h later. (C) Polymers do not affect IFNα production post VSV stimulation. pDCs were generated from bone marrow cells of WT animals in the presence of Flt-3L for 10 days. Cells were then stimulated with live VSV at an MOI of 1 in the presence of 20 µg/mL PAMAM-G3. Supernatants were analyzed for the presence of IFNα by ELISA 24 h later. Data are presented as mean+/− SD and are representative of at least 3 independent experiments. n = 9 mice per group. (D) Polymers do not affect IL6 production post VSV stimulation as compared to TLR inhibitors. DCs were generated form bone marrow cells of WT animals cultured in the presence of IL4 and GMCSF for 7 days. Cells were then stimulated with live VSV at an MOI of 1 in the presence of 20 µg/mL PAMAM-G3, CLI-095 and IRS954. Supernatants were analyzed for the presence of IL6 by ELISA 24 h later. Data are presented as mean+/− SD and are representative of at least 3 independent experiments. n = 9 mice per group. *p<0.05.

We have demonstrated that PAMAM-G3 is capable of neutralizing the inflammatory effects induced by the TLR9 agonist CpG. Since, pro-inflammatory self nucleic acids are recognized by endosomal TLRs that are thought to also respond to viral RNA and DNA, we evaluated whether PAMAM-G3 would inhibit immune cell activation by nucleic acid containing viruses [3], [24], [25]. We infected BMDCs and plasmacytoid DCs with vesicular stomatis virus (VSV) and vaccinia virus (a prototype RNA virus, and DNA virus respectively) in the presence of PAMAM-G3 dendrimer. BMDCs produce pro-inflammatory cytokines including TNFα and IL6 upon viral infection. Both VSV and vaccinia virus have been shown to activate TLRs, including nucleic acid sensing TLRs, and trigger an inflammatory response. pDCs play an important role in viral immunity by producing large amounts of the signature cytokine IFNα upon viral infection [30]. In our study, cell supernatants were analyzed by ELISA for the presence of inflammatory cytokines IL-6 (BMDCs) and IFNα (pDCs). Neither IL-6 nor IFNα levels were decreased in virally infected cells treated with PAMAM-G3 as compared to infected BMDCs or pDCs without PAMAM-G3 treatment (Figure 5A–C). These data demonstrate that PAMAM-G3 does not interfere with the cell’s ability to detect and respond to two prototype RNA and DNA encapsulated viral pathogens. Together these observations suggest that NASPs can be used as potential therapeutic agents to bind accessible, extracellular self-nucleic acids without interfering with normal immune responses to viral pathogens.

To determine if this mechanism of inhibition, targeting the agonist, is fundamentally different from direct inhibition of TLR receptors themself, we next compared PAMAM-G3 to a direct TLR7/9 inhibitor and a TLR4 inhibitor. As previously reported [31], [32], we observed that agents which inhibit directly target and inhibit TLR7/9 and TLR4 resulted in reduced cytokine production by DCs following viral challenge. This result clearly demonstrates that TLR7/9 are important receptors for mounting an anti-VSV response. By striking contrast, the NASP PAMAM-G3 had no effect on cytokine production post viral challenge (figure 5D) even though this NASP inhibits CpG DNA activation of TLR9. These data demonstrate that NASPs act through a different mechanism than traditional therapeutic agents under development that directly target the TLRs. This difference may represent significant advantage for the treatment of autoimmune disorders because NASPs only inhibit activation of TLRs by accessible nucleic acid and do not prevent immune cell recognition of shielded nucleic acids, such as those present in viruses.

## Discussion

We have previously demonstrated that NASPs bind an array of nucleic acids and block activation of nucleic acid-sensing endosomal TLRs [26]. In this study, we show that NASPs do not interfere with normal immune-cell activation that is independent of endosomal TLRs. Moreover our studies demonstrate that despite their ability to block activation of endosomal TLRs by extracellular nucleic acids that are accessible and not encapsulated, NASPs do not inhibit virus triggered immune responses. Thus through their unique mechanism of action, NASPs appear to be potentially useful agents to safely and effectively combat aseptic inflammatory disorders.

Endosomal TLRs 3, 7 and 9 can be activated by endogenous nucleic acid ligands released from dead or dying cells [10], [33], [34]. Moreover, these receptors have been shown to play an important role in the development of autoimmune disorders [23], [35], [36]. Studies of mice deficient in one or several of the TLR genes have shown that these receptors regulate development of inflammatory disorders [37]. TLR3 plays a significant role in the induction of IFN genes upon activation by its dsRNA ligands [38]. Although a direct role has not been attributed to TLR3 in development of SLE, its role in NFkB induction and production of IFNs would suggest that this TLR is implicated in SLE onset. In addition, MRL/lpr lupus prone mice lacking endosomal TLR7 display ameliorated signs of disease [39]. Similarly TLR9 is important in controlling production of autoantibodies during disease development [17]. Together TLR7 and 9 promote SLE progression and further emphasize the importance of nucleic acid signaling in autoimmune disease development. Additionally, lupus prone mice lacking the downstream TLR signaling adaptor MyD88 show improved disease severity similarly to the mice lacking TRL7 and 9 [23]. Although these results point to an important role for TLR7 and 9 in SLE development, it is important to note that mice deficient in TLR7/9 as well as MyD88 are not free of autoimmune disease symptoms [23]. Thus suggesting that certain aspects of inflammatory disorders and autoimmune diseases could be attributed to other nucleic acid sensing receptors such as RIG-1, Mda5 and AIM2 [40]–[43].

Current therapeutic strategies, such as systemic administration of IRS954, have focused on blocking signaling through TLR7 and 9 receptors [22]. Although somewhat effective, these therapeutic approaches have many drawbacks. IRS954 only targets a specific number of nucleic acid receptors known to be involved in autoimmunity, thus still permitting other receptors to contribute to disease development. Moreover, as we have observed (Figure 5D) and as has been previously reported, direct blocking of TLR7 and 9 proteins results in impaired viral recognition by cells expressing these receptors [24], [30]. This could result in long-term immune suppression. By contrast, in our study we demonstrate that NASPs do not affect cell stimulation by viruses such as VSV and VV, while direct TLR7/9 protein inhibition via IRS954 does (Figure 5D). Thus NASPs have a property that may prove to be valuable in the development of safer anti-inflammatory agents that block aberrant cell activation while leaving normal immune responses intact.

Our work focused on two different cell types that are known to be important players in autoimmune and inflammatory disease development, B cells and DCs. B cells from the C57BL/6 mouse spleens did not produce cytokines and antibody when stimulated with CpG DNA, a freely accessible nucleic acid, in the presence of NASPs. In contrast, the B cell responses were intact in the presence of a TLR4 agonist. The effects of NASPs extend beyond controlling cytokine production, thus opening new avenues to target multiple aspects of autoimmunity and inflammation such as aberrant cell proliferation and autoantibody production. Proliferation is also a key aspect of the germinal center reaction where high affinity antibodies are produced.

We next studied another cell type, which plays a critical role in autoimmune disease development. We evaluated the ability of DCs to stimulate T cells in an antigen dependent fashion in the presence of NASPs. In this case the stimulation was nucleic acid TLR independent. Our data demonstrate that DCs were able to stimulate T-cell proliferation and cytokine production in the presence of the NASPs CDP and PAMAM thus demonstrating that these compounds do not affect DC functions that are independent of TLRs.

Our studies also focused on evaluating whether the unique mechanism of action of NASPs may allow immune responses triggered by encapsulated nucleic acids in viruses to take place even though NASPs inhibit such activation by freely accessible extracellular DNA and RNA. In this study we clearly demonstrate that in sharp contrast to agents that directly bind and inhibit TLRs, NASPs do not affect cell activation following viral infection. Future studies will focus on examining the role of NASPs in activation of immune cells *in vivo* by nucleic acid-containing immune complexes. These studies will allow us to further define the mechanism by which NASPs function. NASPs may also play an important role in treating autoimmune disorders caused by deficiencies in nucleases such as DNase, RNase and Trex1 [44]–[46].

Studies presented here underscore how the mechanism employed by NASPs to limit inflammation, scavenging of accessible extracellular nucleic acids, represents a novel and potentially safer strategy to treat autoimmune and inflammatory disorders without compromising an individual’s ability to combat pathogenic viruses.

## Supporting Information

Figure S1 **Optimal polymer concentration for blocking cytokine expression post TLR9 stimulation.** DCs were derived as previously described. DCs were then cultured in the presence of CpG as well as varying doses of each polymer (HDMBr, CDP and PAMAM). Cytokine production was assessed at 18 hrs by ELISA. Data are representative of 3 independent experiments.
(TIF)
