# Supplementary figures and images for: Nucleic Acid Scavenging Polymers Inhibit Extracellular DNA-Mediated Innate Immune Activation without Inhibiting Anti-Viral Responses
Source: PLoS One. 2013 Jul 23;8(7):e69413. doi: 10.1371/journal.pone.0069413 (PMC3720614; doi:10.1371/journal.pone.0069413)

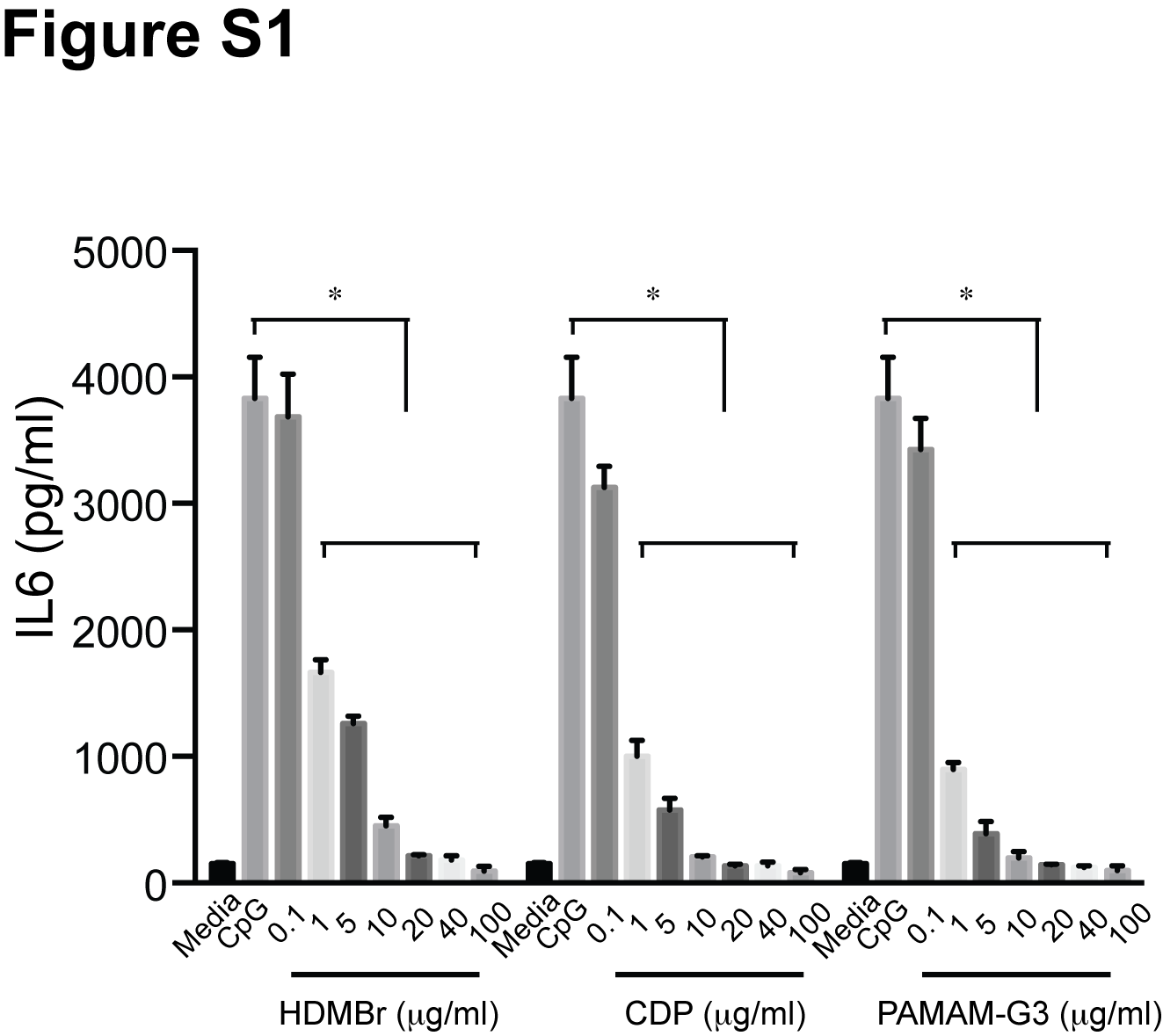

Supplement: Figure S1 — Optimal polymer concentration for blocking cytokine expression post TLR9 stimulation. DCs were derived as previously described. DCs were then cultured in the presence of CpG as well as varying doses of each polymer (HDMBr, CDP and PAMAM). Cytokine production was assessed at 18 hrs by ELISA. Data are representative of 3 independent experiments. (TIF) [file pone.0069413.s001.tif]
